# Percutaneous embolization: a viable treatment option for varicocele

**DOI:** 10.1186/2051-4190-24-10

**Published:** 2014-06-02

**Authors:** Ranjith Ramasamy

**Affiliations:** Department of Urology, Baylor College of Medicine, Houston, Texas 77030 USA

There are several options in the treatment of varicocele. Surgical repair either by open or microsurgical approach, laparoscopy, or through percutaneous embolization of the internal spermatic vein have been used to treat varicocele [1]. Regardless of the chosen technique, the ultimate goal relies on the occlusion of the dilated veins that drain the testis. Percutaneous embolization offers a rapid recovery and can be successfully accomplished in approximately 90% of attempts. However, the technique demands interventional radiologic expertise and has potential serious complications, including vascular perforation, coil migration, and thrombosis of pampiniform plexus [2].

Prasivoravong et al. [3] report results from a case series of men undergoing varicocele embolization. They evaluated men with grade III unilateral varicocele and identified improvements in sperm morphology. Percutaneous embolization can be a valuable treatment option for recurrences after surgery since patients may not want to undergo another operation and surgeons may not feel comfortable with artery and lymphatic sparing technique. Percutaneous approach can offer a viable treatment approach for men complaining of scrotal pain (orchialgia) from varicocele because inflammation and scarring from operation may contribute to additional pain.

Further studies are necessary to determine the benefit of percutaneous embolization in men with bilateral and lower grades of varicocele. Additional comparative studies comparing the different treatment options for varicocele can help determine the best treatment option for men that present with varicocele-associated infertility.
